# Transcriptomic and metabolomic dissection of skeletal muscle of crossbred Chongming white goats with different meat production performance

**DOI:** 10.1186/s12864-024-10304-3

**Published:** 2024-05-04

**Authors:** Yuexia Lin, Lingwei Sun, Yuhua Lv, Rongrong Liao, Keqing Zhang, Jinyong Zhou, Shushan Zhang, Jiehuan Xu, Mengqian He, Caifeng Wu, Defu Zhang, Xiaohui Shen, Jianjun Dai, Jun Gao

**Affiliations:** 1 Institute of Animal Husbandry and Veterinary Science, Shanghai Academy of Agricultural Sciences, Shanghai, 201106 China; 2 Shanghai Municipal Key Laboratory of Agri-Genetics and Breeding, Shanghai, 201106 China; 3 Key Laboratory of Livestock and Poultry Resources Evaluation and Utilization, Ministry of Agriculture and Rural Affairs, Shanghai, 201106 China; 4 Shanghai Academy of Agricultural Sciences, Shanghai, 201106 China

**Keywords:** RNA-seq, Differentially expressed gene, Liquid chromatography-mass spectrometry, Goat meat development

## Abstract

**Background:**

The transcriptome and metabolome dissection of the skeletal muscle of high- and low- growing individuals from a crossbred population of the indigenous Chongming white goat and the Boer goat were performed to discover the potential functional differentially expressed genes (DEGs) and differential expression metabolites (DEMs).

**Results:**

A total of 2812 DEGs were detected in 6 groups at three time stages (3,6,12 Month) in skeletal muscle using the RNA-seq method. A DEGs set containing seven muscle function related genes (*TNNT1*, *TNNC1*, *TNNI1*, *MYBPC2*, *MYL2*, *MHY7*, and *CSRP3*) was discovered, and their expression tended to increase as goat muscle development progressed. Seven DEGs (*TNNT1*, *FABP3*, *TPM3*, *DES*, *PPP1R27*, *RCAN1*, *LMOD2*) in the skeletal muscle of goats in the fast-growing and slow-growing groups was verified their expression difference by reverse transcription-quantitative polymerase chain reaction. Further, through the Liquid chromatography-mass spectrometry (LC-MS) approach, a total of 183 DEMs in various groups of the muscle samples and these DEMs such as Queuine and Keto-PGF1α, which demonstrated different abundance between the goat fast-growing group and slow-growing group. Through weighted correlation network analysis (WGCNA), the study correlated the DEGs with the DEMs and identified 4 DEGs modules associated with 18 metabolites.

**Conclusion:**

This study benefits to dissection candidate genes and regulatory networks related to goat meat production performance, and the joint analysis of transcriptomic and metabolomic data provided insights into the study of goat muscle development.

**Supplementary Information:**

The online version contains supplementary material available at 10.1186/s12864-024-10304-3.

## Background

Domestic goat (*Capra hircus*) plays key roles in meat and milk production. In recent years, China’s meat goat industry has developed rapidly. Compared with other domestic animals, goat meat has more protein and lower fat and cholesterol contents [1]. To our knowledge, animal meat production and quality are controlled by both genetic and environment factors and studies have shown that skeletal muscle accounts for almost 40% of body weight [2, 3]. The Chongming white goat (CM) is an indigenous breed distributed in Chongming Island, Shanghai. In terms of species classification, it belongs to the Yangtze River Delta white goat, and the species is recognized as one of the breeds on the Conservation List of Livestock and Poultry Genetic Resources (Goats) in China and is listed in the Food and Agriculture Organization (FAO) Domestic Animal Diversity Information System (DAD-IS, http://dad.fao.org/). CM goat is white in color, relatively small size, and has excellent reproductive performance, but relatively low meat production performance. For many years, our research team has been working to improve its meat yield and has bred crossbreeds with Boer goats. We found that some of the progenies were closer to the CM in terms of growth performance, while some of the progeny had a greater increase in growth performance.

Currently, The RNA sequencing (RNA-seq) technology has developed rapidly, enabling the analysis of differential expression for transcriptomes in many fields including analyze genetic mechanisms underlying skeletal muscle growth and development in domestic animals [4, 5]. Shen *et al* [5] performed RNA-Seq comparative transcriptome analysis of Longissimus dorsi muscle (L) from two Chinese goat breeds (Liaoning cashmere and Ziwuling black) goats with different meat production performance. RNA‑Seq method have also applied to explore the influence of stress on meat quality in Spanish goats [6], as well as miRNA and mRNA Co-regulate muscle differentiation in fetal Leizhou goats [2]. Over the past decades, a range of functional genes and signaling pathways regulating skeletal muscle development and growth in farm animals have been explored [7]. The myostatin (MSTN) plays an important role in muscle development and a mutation on MSTN creates a target site leading to muscle hypertrophy in Texel sheep [8, 9]. Skeletal muscle transcriptomic analysis of sheep from five Spanish meat breeds indicates that a substantial proportion (51–67%) of the transcriptional output of the ovine skeletal muscle is contributed by a few hundred of genes which are mainly involved in muscular contraction, metabolism, calcium transport and energy homeostasis [10]. The dynamic transcriptome of skeletal muscle development in sheep, and the transcriptome of the transformation of fast and slow muscles were explored in recent years using weighted correlation network analysis (WGCNA) and allele-specific expression analysis [11]. Since the gene expression has tissue-specific and spatiotemporal effects, many of the regulate genes and gene networks in goat growth and meat production process are still not fully elucidated.

Untargeted metabolomics is becoming increasingly common in livestock animal studies. The liquid chromatography-mass spectrometry technology (LC-MS) not only achieves excellent chromatographic separation by increasing the number of peaks detected but also allows exact measurement of the mass of metabolites with higher sensitivity, accuracy, and precision. These characteristics may be useful for efficient subsequent structural elucidation in a metabonomic study. Gao *et al* [12]. identified the Metabolomics changes in L muscle of Finishing pigs following heat stress through LC-MS-Based metabolomics method. Jia et al applied also applied this method to reveal the metabolite dynamic changes during irradiation of goat meat [13]. Kong *et al* combined transcriptomics and metabolomics to reveal improved performance of Hu sheep on hybridization with Southdown sheep [14]. Exploring the dynamics of muscle molecule metabolic markers among goat populations with different growth rates will be helpful to dissection the molecular mechanism of “gene-protein-metabolism-phenotype” related to complex traits such as meat production. There are also studies combined transcriptome and metabolome analysis reveals breed-specific regulatory mechanisms in Dorper and Tan sheep [15]. Chen et al revealed transcriptomes and metabolomes of the longissimus dorsi muscle of the F1 generation of hybrid sheep populations were studied to provide insight into the key genes and metabolites involved in muscle growth and meat quality [16].

In this study, we selected several groups of individuals at different growing stages (3, 6, and 12 months of age) from the crossbred Chongming white goats and compared their Longissimus dorsi muscle (L) and Semitendinosus muscle (T) by RNA-seq and untargeted metabolomic analysis of the corresponding muscle tissue samples also be performed, to explore genes and metabolic markers associated with meat production performance in goats.

## Results

### The results of identification of differentially expressed genes (DEGs)

**Fig. 1:**
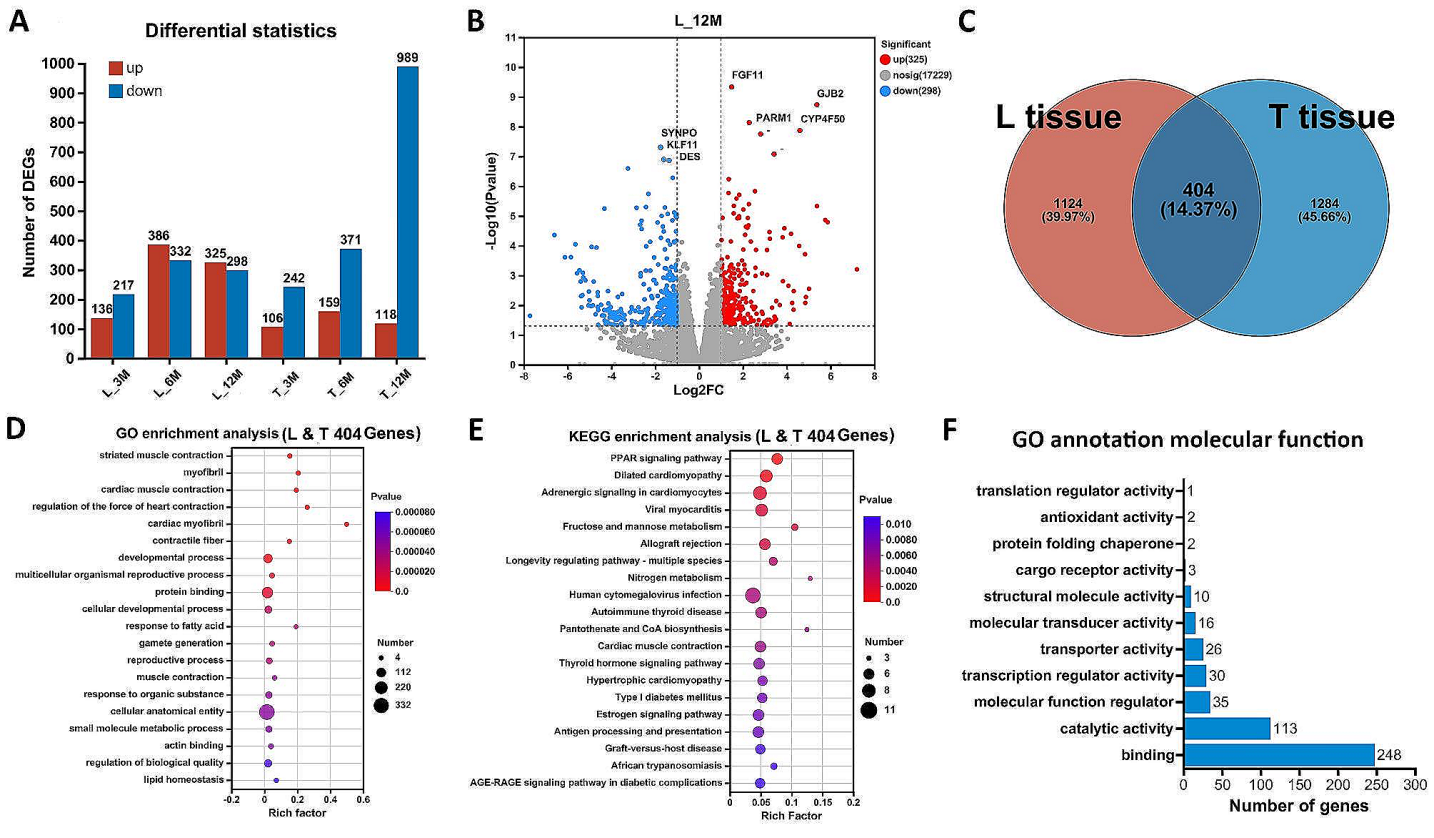
Statistics among groups of differentially expressed genes. **A**. The horizontal coordinates represent the differential comparison groups, and the vertical coordinates represent the corresponding number of up- and down-regulated genes. **B**. The volcano plots showing DEGs in the Longissimus dorsi muscle of the fast-growth and slow-growth groups at 12 months of age. **C**. Venn diagram of DEGs identified among tissues. **D** and **E**. The GO/KEGG enrichment analysis of 404 DEGs shared in L and T muscle tissues. Number indicates the number of genes enriched to the term and the plot demonstrated the most significant Top20 categories with *p* value < 0.05. **F**. The GO molecular function analysis of these 404 DEGs

A total of 2812 DEGs were identified in the L and T muscles tissues of the slow- and fast-growing group goats at 3, 6 and 12 months of age (Fig. 1_A and Supplementary DATA_S1), e.g., a total of 325 genes with up-regulated expression and 298 genes with down-regulated expression were identified between the fast and slow growing groups in the L muscle of goats at 12 months of age (Fig. 1_B). Since it is slow-growing group vs. fast-growing group, a down-regulated gene means that the expression of the gene in the slow-growing group is lower than that in the fast-growing group. Statistics showed that 1528 and 1688 DEGs were identified in the L and T muscles at the three time periods, respectively, with 404 DEGs overlapped (Fig. 1_C).

The GO enrichment analysis of these 404 DEGs showed that the terms of biological progress such as striated muscle contraction (GO:0006941), myofibril (GO:0030016), cardiac muscle contraction (GO:0060048), and developmental process (GO:0032502) were significantly enriched (Fig. 1_D). The KEGG results demonstrated that the important PPAR signaling pathway for fatty acid metabolism and several cardiac muscle related pathways such as Cardiac muscle contraction, Dilated cardiomyopathy, and Adrenergic signaling in cardiomyocytes were enriched (Fig. 1_E). The GO annotation results indicated that 248 of these 404 genes have the molecular function of binding (GO:0005488), 113 genes have catalytic activity (GO:0003824) and 35 genes have the molecular function of regulator (GO:0098772) function (Fig. 1_F).

**Fig. 2:**
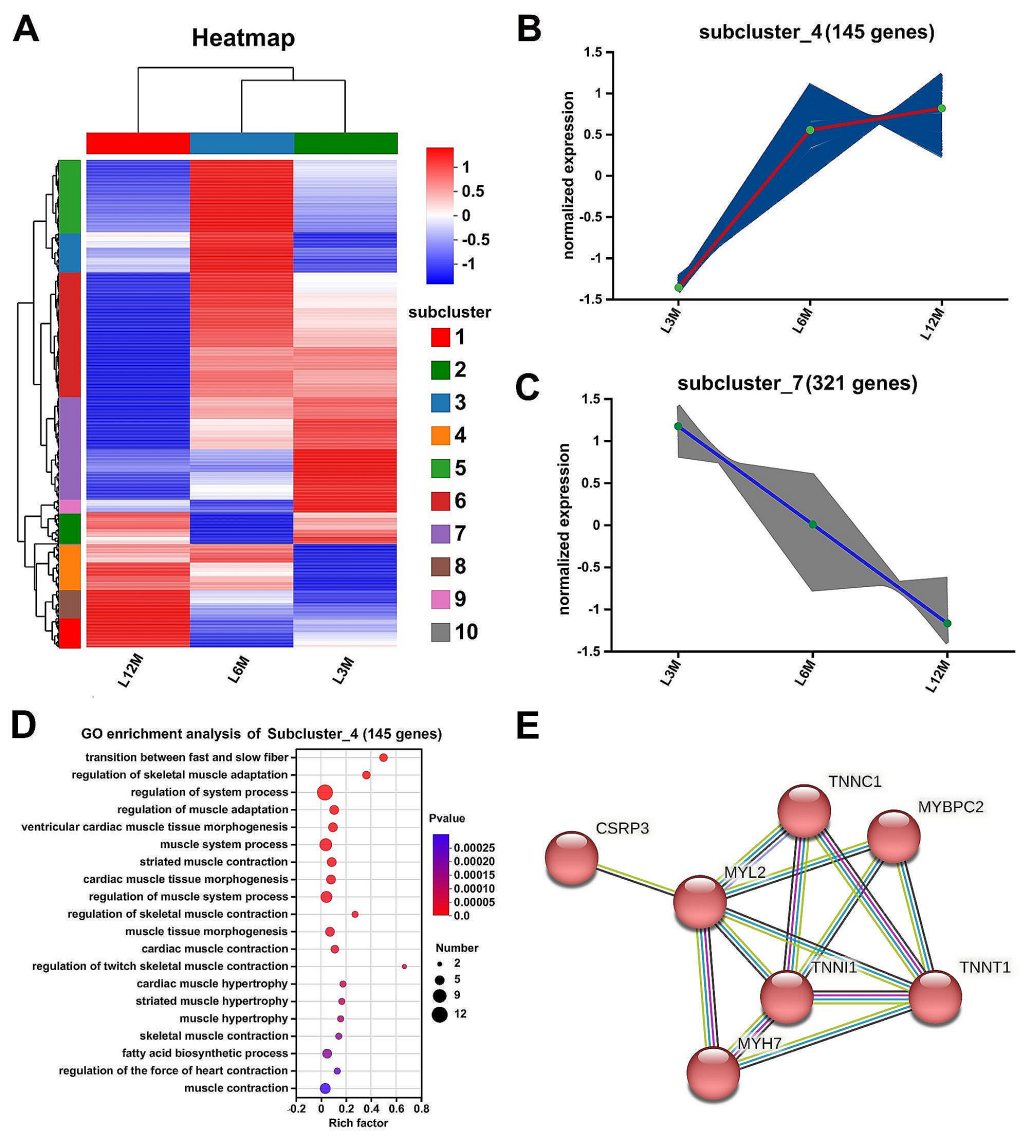
Differentially expressed genes associated with the growth porcess in the L muscle. **A**. Cluster analysis of differentially expressed genes in the L (Longissimus dorsi) muscle. **B**. Sucluster_4 contained 145 genes, and their expression showed an increase trend with the number of months of age of the goats. **C**. Sucluster_7 contained 321 genes, and their expression showed a decrease trend with the number of months of age of the goats. **D** The GO enrichment analyses performed on the Sucluster_4 contained 145 genes. **E**. A PPI subnetwork containing 7 genes related to muscle function was identified from subcluster_4 gene set

Through the cluster analysis of 1528 DEGs in the L groups, a subcluster_4 contained 145 genes showed an increasing trend in expression during growth and development at 3 months of age, 6 months of age, and 12 months of age (Fig. 2_A and Fig. 2_B), and similarly the expression trend of 321 genes in the subcluster _7 gene set of 321 genes showed the opposite trend of continuous decrease in expression (Fig. 2_A and Fig. 2_C). The results of these two gene sets showed that the 145 genes showing an increasing trend, and the genes in this set seemed to be more related to muscle function, because several GO biological process terms (Fig. 2_D) enriched in the muscle related terms (GO:0014883 transition between fast and slow fiber, GO:0014733: regulation of skeletal muscle adaptation, GO:0003012: muscle system process, and GO:0006941: striated muscle contraction, etc.).

### The DEGs associated with skeletal muscle developmental stages

Furthermore, the PPI network analysis indicated a sub-network (Fig. 2_E) of 7 genes including *TNNT1*, *TNNC1*, *TNNI1, MYBPC2*, *MYL2*, *MHY7*, and *CSRP3* in a collection of 145 genes, whose expression tended to increase with development process. These seven genes showed enriched in the muscle contraction term (GO:0006936), Three genes (*TNNT1*, *TNNC1*, and *TNNI1*) related to the troponin complex (GO:0005861). The *MHY7* also related to the actomyosin (GO:0042641) and contractile actin filament bundle (GO:0032432). It indicated that the co-regulation of these several DEGs and their networks crucial to meat production performance during the growing period of CM goats.

### The DEGs between the fast- and slow-growing groups

**Fig. 3:**
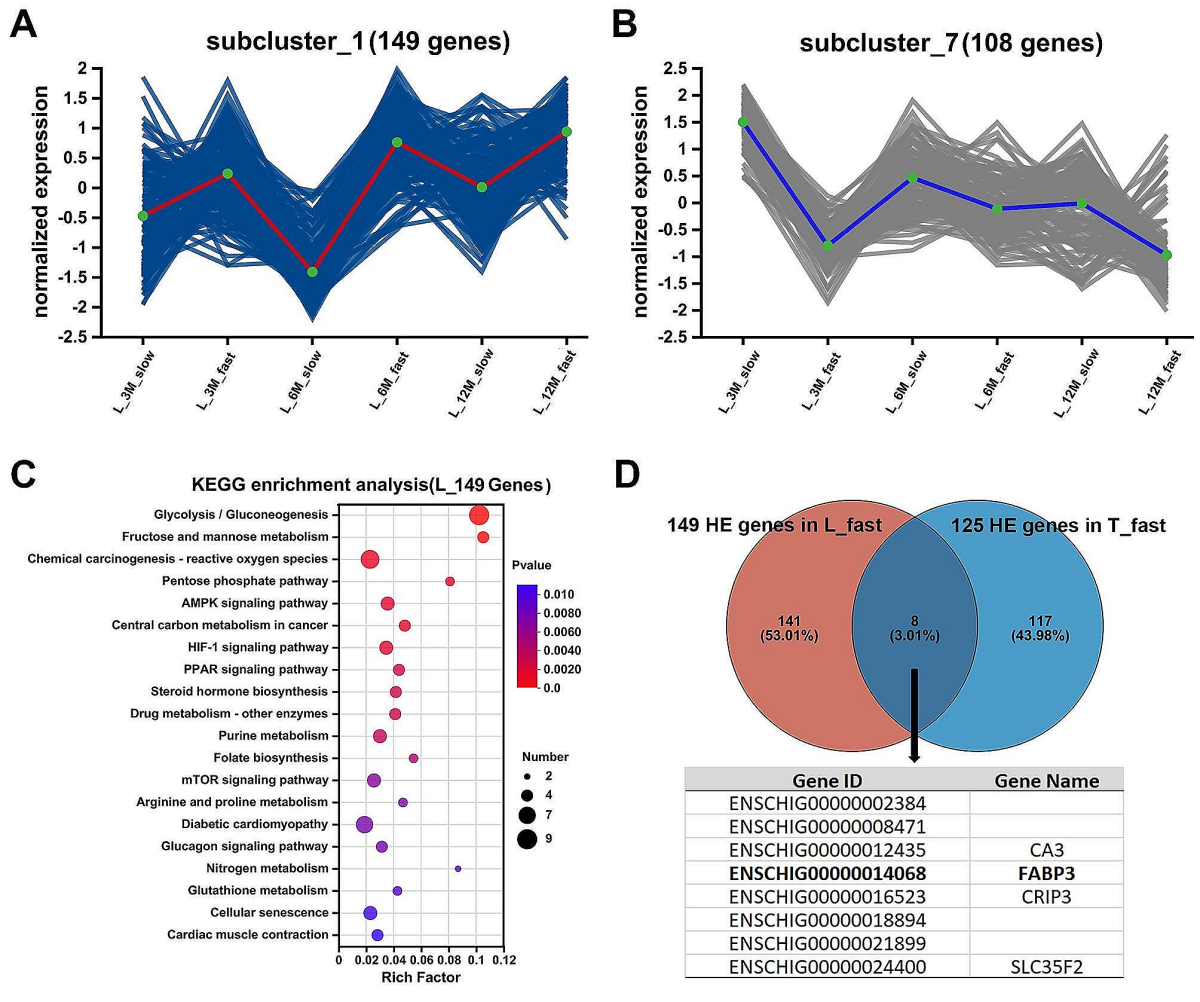
Identification of gene sets between fast- and slow-growing groups. **A**. 149 genes in the gene set of subcluster_1, and the normalized expression of them are higher in the fast-growing group. **B**. 108 genes contained in subcluster_7 had higher expression in the slow-growing group. **C**. The KEGG pathway analysis of the 149 genes in the subcluster_1 sets. **D**. Two gene sets in the fast-growing group of Longissimus dorsi (L) and Semitendinosus (T) muscle revealed eight overlapping highly expressed (HE) genes including the fatty acid binding protein 3 (FABP3) involving the PPAR signaling pathway

On the other hand, through gene clustering, we also uncovered the DEGs sets between the fast- and slow-growing groups, as can be seen in Fig. 3, there are 149 genes in the gene set of subcluster_1, and the normalized expression of them are higher in the fast-growing group than in the slow-growing group at the same time point. Whereas the 108 genes contained in subcluster_7 (Fig. 3_A), on the contrary, had higher expression in the slow-growing group than in the fast-growing group at the same time point (Fig. 3_B). KEGG pathway analysis showed that the 149 genes with relatively high expression in the fast-growing group were mainly enriched in the pathways such as Glycolysis/Gluconeogenesis, AMPK signaling pathway, PPAR signaling, and Diabetic cardiomyopathy pathway (Fig. 3_C). The genes with relatively high expression in the slow-growing group were mainly enriched in Chemokine signaling pathway, FoxO signaling pathway, AMPK signaling pathway (Fig. 3_D), etc.

Similarly, we performed the same cluster analysis and comparison for T muscle, and we identified and obtained a gene set consisting of 125 genes that were relatively highly expressed in the fast-growing group and 8 genes that were relatively highly expressed in the slow-growing group (Supplementary DATA_S1), and for the 2 gene sets that were highly expressed in the fast-growing group of L and T muscle tissues (149 and genes and 125 genes) were compared, and 8 genes were found to be overlapping in the two gene sets, including genes such as fatty acid binding protein 3 (*FABP3*), carbonic anhydrase 3 (*CA3*), and cysteine rich protein 3 (*CRIP3*).

**Fig. 4:**
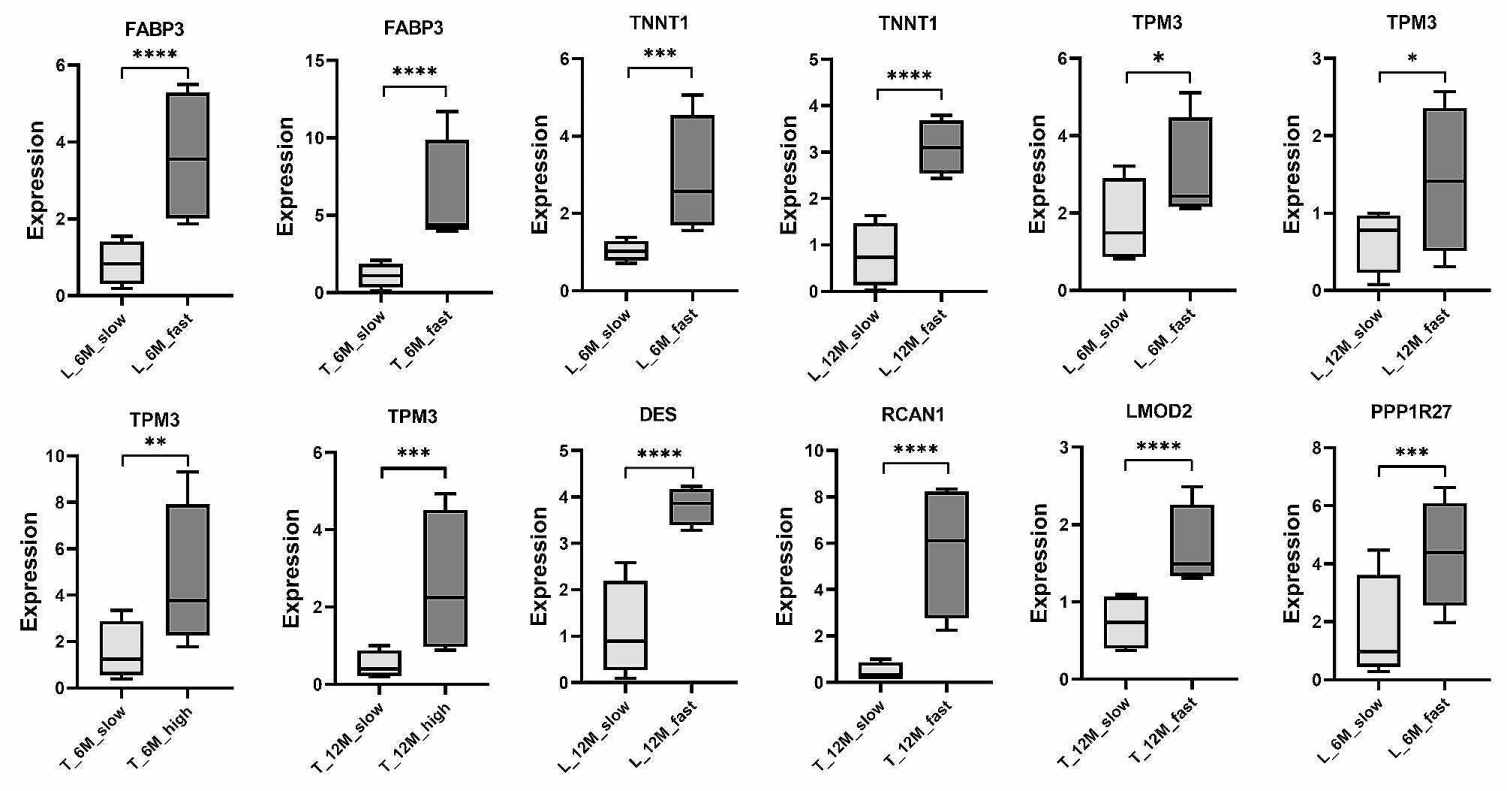
Reverse transcription-quantitative PCR to verify the expression of relevant DEGs. The horizontal coordinate is the name of the sample subgroup, and the vertical coordinates represent the relative expression of genes. The asterisks represent significance, * represents the *p* value < 0.05, ** represents the *p* value < 0.01, *** represents the *p* value < 0.001, **** represents the *p* value < 0.0001

Notably the functional annotation of these genes revealed that *FABP3* as an important fatty acid-binding protein (NR description) participates in the PPAR signaling pathway with the function of lipid transport and metabolism (COG Functional Categories). Combined with the above bioinformatics analysis, we screened seven DEGs (*TNNT1*, *FABP3*, *TPM3*, *DES*, *RCAN1*, *LMOD2*, and *PPP1R27*) with high relative expression in the muscle differential RNA-seq group and verified their expression by RT-qPCR experiments (Fig. 4). The results were in complete agreement with the trend of RNA-seq expression differences.

## Results of the analysis of DEMs in different parts of skeletal muscle

**Fig. 5:**
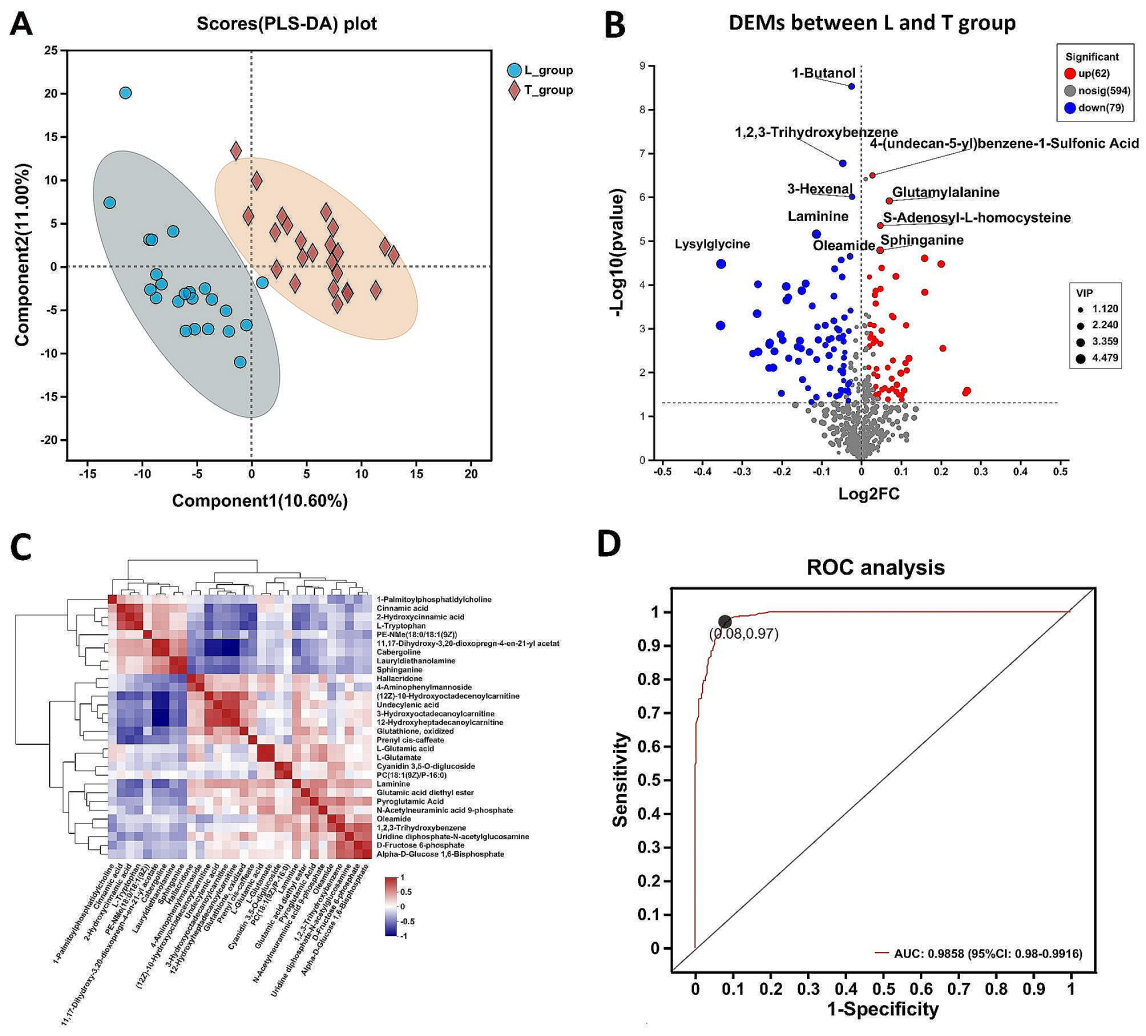
The different expression metabolites between L and T muscle. **A**.PLS-DA analysis of metabolomics between L and T samples. **B**. The volcano plots showing DEMs between the Longissimus dorsi (L) and Semitendinosus (T) muscle. **C**. The heatmap illustrates the correlation between the top 30 metabolites in abundance among these 141 DEMs. **D**. Receiver Operating Characteristic (ROC) analysis of combine of the 141 DEMs

In this study, the muscle samples from the above RNA-seq test were also analyzed for untargeted metabolites. The different expression metabolites (DEMs) analysis performed between 24 L muscle samples and 24 T muscle samples. The PLS-DA results showed significant differences between the metabolites of the L and T muscles of goats (Fig. 5_A). A total of 141 DEMs with names were identified and obtained, of which 62 were up-regulated and 79 were down-regulated (Fig. 5_B and Supplementary DATA_S2). The down-regulation represented a significantly lower abundance of the metabolite in the L muscle compared to the T muscle. For example, the volcano plots show significantly lower levels of 1-Butanol in the L muscle group than in the T muscle group (Fig. 5_B and Fig. 5_C). The VIP values for each of these significant metabolites were also obtained by analyzing the magnitude of the VIP values, with Lysylglycine having the highest VIP value (Fig. 5_B and Fig. 5_D), indicating that this metabolite contributed the most to the classification of the samples between the L and T muscles.

The study also calculated the correlation between the top 30 most abundant metabolites in these 141 DEMS (Fig. 5_C). The Receiver Operating Characteristic (ROC) analysis also demonstrated the accurate predictive value of these 141 DEMs for the differentiation of L and T muscle groups (AUC = 0.9858, Fig. 5_D).

### DEMs between fast- and slow-growing groups

**Fig. 6:**
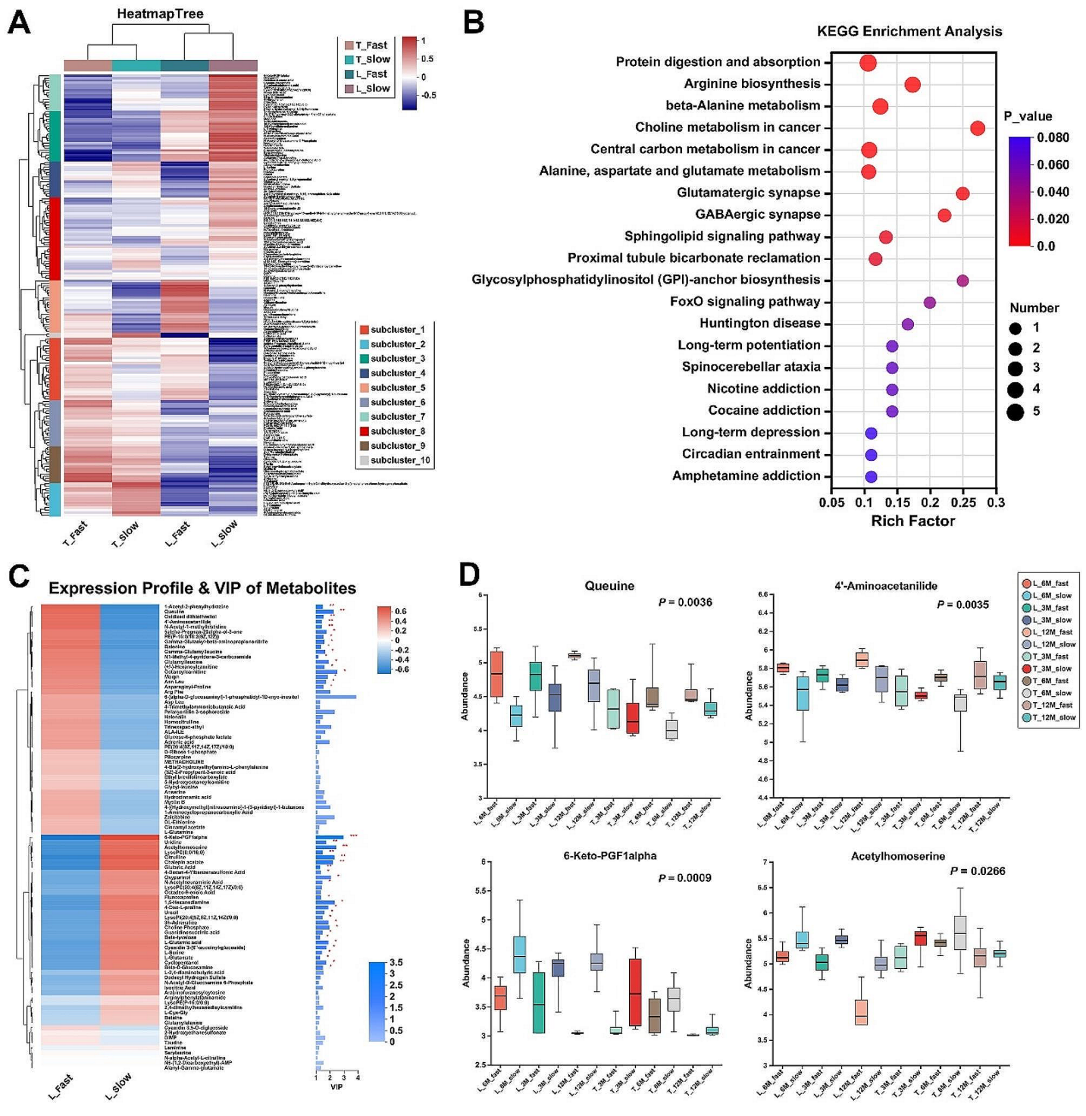
Different expression metabolites identified between fast- and slow-growing group. **A**. Cluster analysis of 183 different expression metabolites (DEMs). **B**. KEGG pathway analysis of 183 DEMs. **C**. The VIP value analysis of 183 DEMs in the L muscle. That contributed most to group separation, showing VIP values, which indicated that these variables had a major contribution to the separation of each group. **D**. Four significant DEMs showing significant differential expression in fast-growing and slow-growing muscles

After studying the metabolites of between L muscle and T muscle, a total of 183 Different expression metabolites (DEMs) were identified in the multiple comparisons of the slow-growing group with the fast-growing group (*p* value < 0.05), and the results of the analysis were placed in Supplementary DATA_S2. Subsequently, these 183 DEMs were clustered in the heatmap (Fig. 6_A), most of the clustered metabolites in Subcluster 5 and Subcluster 1 were found to be highly expressed in the fast-growing group, both in the L and T muscles. In contrast, The DEMs in Subcluster 7, Subcluster 3, Subcluster 4, and Subcluster 10 were relatively highly expressed in the slow-growing group. These 183 DEMs were significantly enriched in several metabolism-related pathways, including Protein digestion and absorption, Arginine biosynthesis, and Glutamatergic synapse (Fig. 6_B).

For further analysis, for example, in Subcluster 5 containing 22 DEMs, Queuine and 4’-Aminoacetanilide were shown to have significant VIP values in the metabolite VIP analysis (Fig. 6_C) and showed higher metabolite abundance in the fast-growing group compared to the slow-growing group at all time points and in muscle tissue (Fig. 6_D). The DEMs in Subcluster 7 with consistently significantly higher abundance in the slow-growing group included 6-Keto-PGF1alpha and Acetylhomoserine (Fig. 6_C and Fig. 6_D).

### The results of weighted correlation network analysis between DEGs and DEMs

**Fig. 7:**
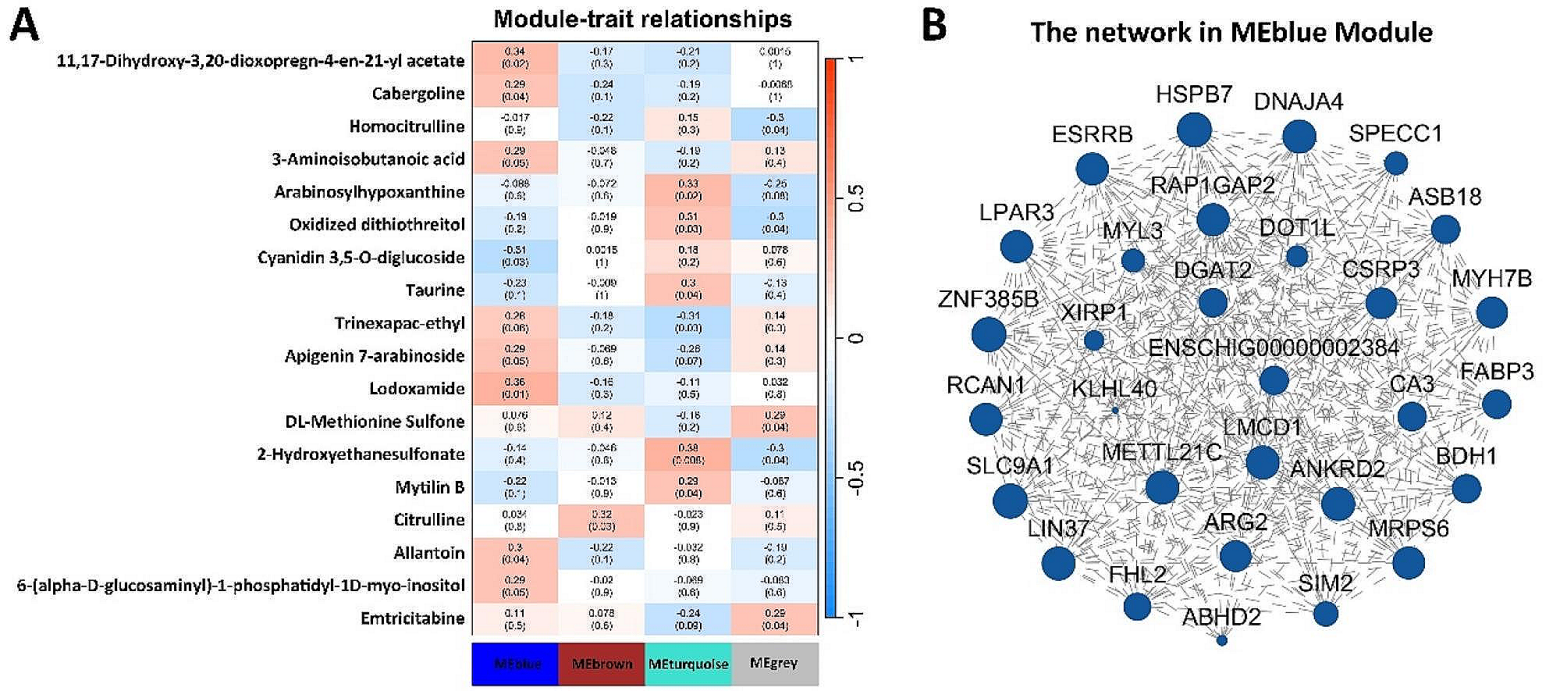
Weighted correlation network analysis between the DEGs and DEMs. **A**. 239 DEGs are assigned into 4 modules (MEblue, MEbrown, MEturquoise, and MEgrey) correlation with 18 DEMs. **B**. The Network analysis of the 30 DEGs with the highest connectivity in MEblue Module

A total of 239 genes were obtained from the 404 differentially expressed genes shared in the L and T groups described above in Fig. 1_C, after removing genes with expression means less than 1 and coefficients of variation less than 0.1. By weighted correlation network analysis (WGCNA), these genes were linked to the above 183 DEMs identified from the multiple comparisons of the slow-growing group with the fast-growing group. A total of four Modul were identified to be correlated with 18 metabolites (Fig. 7_A), with the gene set of the MEblue module containing 68 genes, 38 genes in MEbrown, 79 genes in MEturquoise, and 54 genes in MEgrey (Supplementary DATA_S2). Among them, MEblue was significantly associated (*P* < 0.05) with 8 out of 18 metabolites. This was followed by MEturquoise, which was significantly associated with 6 metabolites. The Network analysis of the 30 genes (Fig. 7B) with the highest connectivity among the 68 genes of MEblue showed several functional genes related to muscle, such as *FABP3*, *RCAN1*, *CSRP3*, *MYH3*, and *MYH7B*. The results of WGCNA also demonstrates the correlation between each DEGs and DEMs, and the most relevant metabolite for FABP3 was found to be Cabergoline (*r* = 0.413, Supplementary DATA_S2).

## Discussion

Animal locomotion, respiratory function, and the pumping motion of the heart all depend on the forces generated by the striated muscle, which includes both skeletal and cardiac muscle. The troponin complex regulates muscle contraction and multiple variants in skeletal troponin encoding genes result in congenital myopathies [17, 18]. In this study, we dissection a gene network comprises of seven genes (*TNNT1*, *TNNC1*, *TNNI1*, *MYBPC2*, *MYL2*, *MHY7*, and *CSRP3*) in the L muscles of goats showed a trend of increased expression during the growth and developmental stages., and this regulatory network related to the troponin complex likely as important regulator on skeletal muscle development during the growth and developmental stages of goats. *TNNT1* encodes the slow skeletal muscle troponin T (ssTnT) which is a vital component of the troponin complex that is integral to Ca2 + regulation of skeletal muscle contraction. Variants in *TNNT1* cause nemaline myopathy 5 in Human, which has a severe phenotype, leading to loss of slow twitch myofibers [17, 19]. In mice, ssTnT deficiency causes atrophy of type I myofibers [20]. Latest study had been identified *TNNT1* gene is the genetic cause of Ovine congenital progressive muscular dystrophy (OCPMD) in the western Australian sheep flock [21]. The over-expression of *TNNI1* significantly enhanced the expression of the skeletal muscle development-related genes in New Zealand white rabbits, and the silencing of *TNNI1* gene significantly reduced expression of these genes. It revealed that *TNNI1* may be a regulator for regulating the expression of muscle development-related genes [22]. In cold-exposed Altay lambs, the gene expressions of *PVALB*, *TNNC1*, *MYL2*, and *ACTC1* related to muscle contraction were higher in muscle tissue of Altay than Hu lambs [23]. *MYBPC2* were also reported as marker genes in muscle tissue, which are mainly involved in glycolysis/gluconeogenesis, AMPK pathway and insulin pathway and related to bovine muscle development [24]. Vitamin C promotes muscle development mediated by the interaction of *CSRP3* with *MyoD* and *MyoG* [25]. Therefore, these seven genes dissected in this study are clearly functionally important for the growth and muscle development of Chongming white goats, providing insights into differences in meat production performance in goats.

*FABP3*, which is involved in the PPAR signaling pathway and is abundantly expressed in the heart and muscle tissues [26]. *FABP3* can directly bind to cellular insoluble free long-chain fatty acids and transport them to the mitochondrion, nucleus, or endoplasmic reticulum for utilization [27]. A study revealed the relationship between *FABP3* and obesity in vivo and the effects of *FABP3* on signal transduction for glucose uptake in skeletal muscle cells in vitro. The level of FABP3 protein in gastrocnemius muscles increased significantly with an increase in body weight and metabolic phenotypes in obese mice, suggesting a close relationship between *FABP3* expression in the muscle and the development of obesity and/or insulin resistance in mice [28]. Several studies have revealed that the *FABP3* gene is involved in muscle development and meat marbling [29, 30]. *FABP3* had been identified as a positive effect on the intramuscular fat content or fat deposition in the muscle of pigs or sheep [31–33]. The evidence including the validation results of RT-qPCR indicated a high correlation between this gene and the growth and meat production performance phenotypes of the goat population, suggesting that this *FABP3* as an important candidate gene for grow period of our crossbred Chongming white goat population.

Tropomyosin (TPM) is a protein family associated with the stabilization and regulation of the actin cytoskeleton [36]. In striated muscle, together with troponin, it enables calcium regulation of striated muscle contraction by blocking (-Ca^2+^) or allowing (+ Ca^2+^) myosin access to its binding site on actin [37, 38]. In mammals, four tropomyosin genes *TPM1*, *TPM2*, *TPM3*, and *TPM4* are known [38]. The unique distribution pattern of *TPM3* adds to the diversity of the tropomyosin family and strongly suggests functional significance for the different striated muscle TM isoforms. A study revealed Muscle weakness in TPM3-myopathy is due to reduced Ca^2+^ -sensitivity and impaired acto-myosin cross-bridge cycling in slow fibers [39]. *TPM3* has high expression in muscle tissues and significant differences in expression were found in skeletal muscle between breeds with different growth performance in several studies [40–43]. Combined with the significant difference in the expression of *TMP3* gene in both muscle tissues of fast-growing and slow-growing groups at 6 and 12 months of age of goats found in this study, it suggests that this gene is closely related to the meat production performance of goats. In this study, RT-qPCR was also utilized to validate several DEGs that may be associated with muscle development in goats, such as desmin (*DES*) is the primary intermediate filament of cardiac, skeletal, and smooth muscle [44]. The leiomodin-2 (*LMOD2*) are critical for specifying thin-filament length in skeletal muscle [45]. The regulator of calcineurin 1 (*RCAN1*) was reported significantly different in L muscles of Leizhou Black goats in postnatal muscle development [2]. The muscle RNA-seq data from the Eonjena Taeyang horses observed that *PPP1R27* shown upregulation at the postexercise period [46]. The protein phosphatase 1 regulatory subunit 27 (*PPP1R27*) also reported as candidate genes of wool-related traits in Sheep [47]. The expression of these gene in the muscle tissues of fast-growing and slow-growing goats in the present study differed significantly and deserves more in-depth elucidation.

In this study, small molecule metabolites of the longissimus dorsi and semitendinosus muscle of goats in the fast-growing and slow-growing groups were investigated using LC-MS technique, and 183 differential metabolites were identified, such as Queuine and 4’-Aminoacetanilide, which were significantly more abundant than those of slow-growing group in all fast-growing groups. Queuine is a hypermodified nucleobase, which is modified to Queuosine [48]. Queuine, as an important micronutrient, derivative made exclusively by eubacteria and salvaged by animal, plant, and fungal species [49]. Some studies have concluded that it is an important “longevity vitamin” that play a dual role in survival and longevity [50]. In the present study, the abundance of Queuine was found to be significantly higher in the muscles of the fast-growing group of goats than in the slow-growing group, and the mechanism by which it contributes to the growth and muscle development of goats as well as other animals remains to be elucidated. Some metabolites, however, showed significantly higher abundance in the muscle of the slow-growing group of goats than in the fast-growing group, such as 6-keto-PGF1α and Acetylhomoserine. Prostaglandin production was measured of sartorius muscle of aged and young adult rhesus monkeys. The results showed a greater percentage of 6-keto-PGF1α production by aged muscle than by young adult muscle [51]. We believe that these metabolites should be further investigated and perhaps applied as candidate metabolite markers for future muscle development in goats. Through WGCNA analysis, we correlated the transcriptome data with the metabolome data, and by analyzing the co-expression network of DEGs and DEMs in each group, we identified a different set of genes associated with 18 metabolites, which opens the possibility of further investigating the associations between these DEGs and DEMs.

Through WGCNA analysis, we identified different gene modules associated with 18 DEMs. Some known muscle-related functional genes (e.g., *FABP3*, *RCAN1*, *CSRP3*, *MYH3*, and *MYH7B*) [2, 25, 28, 29, 52, 53] among the 68 genes in MEblue module, which was significantly associated with eight differential metabolites (*P* < 0.05). The genes in this module may be instructive for the discovery of a regulatory network for skeletal muscle development in goats. There are seven differential metabolites that show significant positive correlation with this gene module (e.g. Lodoxamide, Allantoin, and Cabergoline) and one negative (Cyanidin 3,5-O-diglucoside). Cabergoline treatment promotes myocardial recovery in peripartum cardiomyopathy [34]. There are also reports of during therapy with Cabergoline, all patients reported a significant improvement of restless legs syndrome [35]. Allantoin significantly increased the expression of myogenetic proteins in skeletal muscle tissues [54]. Ischaemic murine skeletal muscle reperfused for 1 h showed much less degranulation of mast cells in mice pretreated with lodoxamide than in saline-treated controls [55]. Although these few muscle differential metabolites identified in this study, it is uncertain whether they can be future biomarkers for identifying fast and slow growing goats. However, they could be important candidate targets for studying the mechanisms of muscle development in goats.

## Conclusions

Through transcriptomics and metabolomics comparison of the skeletal muscles of fast- and slow-growing groups at different stages of the crossbred Chongming white goats. Some functional genes (such as *FABP3* and *TPM3*) and gene regulatory networks (*TNNT1*, *TNNC1*, *TNNI1*, *MYBPC2*, *MYL2*, *MHY7*, and *CSRP3*) that closely related to the muscle development of goats were explored. The study also identified significant metabolites (such as Queasiness and keto-PGF1α) in the muscles of fast- and slow-growing goats. The study also utilized WGCNA to mine relevant gene modules and differential metabolic markers associated with them. All these provide insights and research basis for the molecular genetic breeding of Chongming white goats.

## Materials and methods

### Sample collection

The samples collected in this study were all from the crossbred Chongming white goats (An improved population in the early years from the crossbreeding of the indigenous CM goat with the Boer goat) that were self-bred farm in our institute. The breeding farm located in Chongming island, Shanghai, China. To avoid interference in litter size and male and female sex, only rams born as double lambs were selected for this study. The body weights and body sizes of these goats were measured at 3, 6 and 12 months after birth, respectively, and each four samples of the fast- and slow-growing group were selected for slaughtering and sampling at each of the three time points. Goats are anesthetized with the proper amount of anesthesia and slaughtered painlessly. For growth and slaughtering performance measurement, we referred to the agricultural industry standard of the People’s Republic of China (Technical Specification for sheep and goat stud productivity testing, NY/T1236-2006). Composition and nutrient levels of goat diets attach in the Supplementary DATA S3. Fasted for 16 h before slaughter, and water was forbidden for 2 h before slaughter, and the goat were weighed. After bloodletting, the whole carcass (including kidneys) was rested for 30 min for carcass weight measurement after removing the fur, the head, the forelimb carpal joints, the hindlimb below the fly joints, and the internal organs. The tissue samples collected included the L muscle on the left side of the back between the 12th and 13th ribs and T muscle of the left hind leg. Immediately after each fresh tissue collection, it was preserved in liquid nitrogen and brought back to the laboratory and stored in a -80 °C refrigerator. The information of tissue samples collected was listed in Table 1. The sample numbers and tissues collected are listed in the Table 1. The Phenotypic measurements such as body weight of these goats are presented in Supplementary DATA_S1.

**Table 1:** The information of *Longissimus dorsi* muscle (L) samples and groups

3 months (3M_group)	3M_Sample_ID	6 months (6M_group)	6M_Sample_ID	12 months (12M_group)	12M_Sample_ID
L_3M_slow	M2112	L_6M_slow	M1247	L_12M_slow	M1127
L_3M_slow	M2144	L_6M_slow	M2068	L_12M_slow	M1172
L_3M_slow	M2195	L_6M_slow	M2171	L_12M_slow	M1196
L_3M_slow	M2192	L_6M_slow	M2176	L_12M_slow	M1194
L_3M_fast	M2120	L_6M_fast	M1153	L_12M_fast	M1152
L_3M_fast	M2145	L_6M_fast	M1261	L_12M_fast	M1165
L_3M_fast	M2229	L_6M_fast	M2011	L_12M_fast	M1177
L_3M_fast	M2266	L_6M_fast	M2071	L_12M_fast	M1228

Note: Skeletal muscle including *Longissimus dorsi* muscle (L) and *Semitendinosus* muscle (T) were collected separately for each sample. The T muscle group information are similar to those in Table 1, except that the initial letter of tissue category changing from L to T

### RNA-seq

RNA-seq was performed on total of 48 tissue samples as mentioned above (24 samples each from L and T tissues). Sequencing experiments were performed using the Illumina Truseq™ RNA sample prep Kit method for library construction. Total RNA was extracted from tissue samples, and the concentration and purity of the extracted RNA were examined using Nanodrop2000, RNA integrity was detected by agarose gel electrophoresis, and RIN values were determined by Agilent2100. A single library was required to have total RNA ≥ 1ug, concentration ≥ 35ng/µL, OD260/280 ≥ 1.8, OD260/230 ≥ 1.0. A-T base pairing with ploy A using magnetic beads with Oligo (dT) allows isolation of mRNA from total RNA. High-throughput sequencing based on the Illumina Novaseq 6000 sequencing platform and performed in Shanghai Majorbio Company (www.majorbio.com), China. Clean data (reads) were mapped to the goat reference genome ARS1 (gca_001704415.1) [56] using HISAT2 v2.2.1 [57]. Transcripts from each sample were assembled using Cufflinks v2.2.1 or StringTie softwarev2.2.0 [58, 59]. The expression levels of the transcripts were quantified using the expression quantification software RSEM v1.3.3 [60, 61], and the quantification index was transcripts per million (TPM).

DEGs were identified based on the fold change (FC) expression of genes that were up-regulated 2-fold and down-regulated 2-fold or more (FC ≧ 2 and FC ≦ 0.5) with a *P*-value < 0.05 between groups using DESeq2 v1.42.1 [62]. The GO (Gene ontology) enrichment analysis of genes in the gene set was performed using the software GOATOOLS v1.1.6 [63], using Fisher’s exact test, and when the *P* value < 0.05.Hierarchical clustering analysis is performed using the R package of fastcluster v1.2.2 [64]. The heatmap clustering is normalized by z-score, which is obtained by converting the TPM values to log10. Protein-Protein Interactions (PPI) [65] was performed on the online STRING v11 (Search Tool for the Retrieval of Interacting Genes/Proteins, https://www.string-db.org/) database. The parameters are set the high score of confidence interaction of 0.7 and Markov Cluster Algorithm (MCL) clustering was used for subnetwork constructionand the inflation parameter used a default setting 3.

Reverse transcription-quantitative PCR (RT-qPCR) analysis was used to identify the seven interest DEGs (*TNNT1*, *FABP3*, *TPM3*, *DES*, *RCAN1*, *LMOD2*, and *PPP1R27*) expression in several skeletal muscle related groups. The *YWHAZ* (Tyrosine 3-monooxygenase/tryptophan 5-monooxygenase activation protein, zeta polypeptide) gene were suggested to accurately measure the true expression levels of target genes during the skeletal muscle of different development stages in goats [66]. The primer information of the above genes is listed in the Supplementary material Table S1. The RT-qPCR were conducted in triplicate using ChamQ SYBR Color qPCR Master Mix (2X) (Vazyme, Nanjing, China) on an ABI7300 Fluorescence Quantitative PCR Instrument (Applied Biosystems, MA, United States). The relative expression levels of these DEGs were analyzed using the 2 ^−△△CT^ method [67].

### Untargeted metabolomics

#### Muscle metabolite extraction

All the 48 muscle samples from the above RNA-seq study were used in this experiment. Muscle Sample $(\sim 50\,{\rm{mg}})$ was added to a 2 mL centrifuge tube and 400 µL of extraction solution (methanol: water = 4:1 (v: v)) containing 0.02 mg/mL of internal standard (L-2-chlorophenylalanine) was used for metabolite extraction. Samples were ground under frozen tissue grinder for 6 min (-10 °C, 50 Hz), followed by low-temperature ultrasonic extraction for 30 min (5 °C, 40 kHz). The samples were left at -20 °C for 30 min, centrifuged for 15 min (4 °C, 13,000 g), and the supernatant was transferred to the injection vial for LC-MS analysis.

#### LC-MS analysis

LC-MS analysis conducted on a Thermo UHPLC-Q Exactive HF-X system equipped with an ACQUITY HSS T3 column (100 mm × 2.1 mm i.d., 1.8 μm; Waters, USA) at Majorbio Bio-Pharm Technology Co. Ltd. (Shanghai, China). The mobile phases consisted of 0.1% formic acid in water: acetonitrile (95:5, v/v) (solvent A) and 0.1% formic acid in acetonitrile: isopropanol: water (47.5:47.5, v/v) (solvent B). The flow rate was 0.40 mL/min, and the column temperature was 40°C. The mass spectrometric data were collected using a Thermo UHPLC-Q Exactive HF-X Mass Spectrometer. The optimal conditions were set as followed: source temperature at 425$^{\circ}{\rm C }$; sheath gas flow rate at 50 arb; Aux gas flow rate at 13 arb; ion-spray voltage floating (ISVF) at -3500 V in negative mode and 3500 V in positive mode, respectively; Normalized collision energy, 20-40-60 V rolling for MS/MS. Full MS resolution was 60,000, and MS/MS resolution was 7500. Data acquisition was performed with the Data Dependent Acquisition (DDA) mode. The detection was carried out over a mass range of 70–1050 m/z. As a part of the system conditioning and quality control process, a pooled quality control sample (QC) was prepared by mixing equal volumes of all samples. It helped to represent the whole sample set, which would be injected at regular intervals in order to monitor the stability of the analysis.

The pretreatment of LC-MS raw data was performed by Progenesis QI v3.0 (Waters Corporation, Milford, USA) software. At the same time, variables with relative standard deviation (RSD) > 30% of the QC samples were deleted and log10 logarithmized to obtain the final data matrix for subsequent analysis. The selection of significantly different metabolites was determined based on the variable weight values (VIP) obtained from the orthogonal Partial Least Squares Discriminant Analysis (OPLS-DA) model and the student’s t-test *p*-value, and metabolites with variable importance in projection value (VIP) > 1, *p* < 0.05 were considered as significantly different metabolites. one-way ANOVA significance test, Benjamini & Hochberg test for *P*-value between groups. Differential metabolites were obtained annotation via the Human Metabolome Database (HMDB v5.0, http://www.hmdb.ca/) [68] and KEGG database v102 (https://www.kegg.jp/kegg/pathway.html) [69].

#### The weighted gene co-expression network analysis (WGCNA)

The DEMs identified from the untargeted metabolome were combined with DEGs in WGCNA analysis [61]. It was performed on normalized counts from DESeq2 of RNA-seq data after removing genes with expression means less than 1 and coefficients of variation less than 0.1. An adjavency matrix was built with a soft thresholding (β value = 8). Calculating module correlations using Pearson’s correlation coefficient. The minimum number of genes/transcripts that make up a module is set to 30. Gene clustering dendrogram was performed with height default cutoff of 0.25. Filter the top 30 nodes of connectivity within a module for analysis and the filtering nodes is greater than 0.02 were analyzed. The WGCNA analysis was performed on the free online platform of majorbio (cloud.majorbio.com) [70].

### Electronic supplementary material

Below is the link to the electronic supplementary material.

Additional file 1: Supplementary DATA_S1: Additional data on goat sample information and RNA-seq sequencing

Additional file 2: Supplementary DATA_S2: The statistics of different expression metabolites (DEMs)

Additional file 3: Supplementary DATA S3: Composition and nutrient levels of goat diets.docx

Additional file 4: Table S1: The primers information of RT-qPCR experiments

## Data Availability

The original contributions presented in the study are publicly available. The RNA-seq data can be found here: NCBI (https://www.ncbi.nlm.nih.gov/), Project with accession numbers of PRJNA1004349. Metabolome raw data are available upon reasonable request by contacting the corresponding author (J.G.).
