# Supplementary material for: Transcriptomic and metabolomic dissection of skeletal muscle of crossbred Chongming white goats with different meat production performance
Source: BMC Genomics. 2024 May 4;25:443. doi: 10.1186/s12864-024-10304-3 (PMC11069289; doi:10.1186/s12864-024-10304-3)
Supplement: Supplementary file 3 — Additional file 3: Supplementary DATA S3: Composition and nutrient levels of goat diets.docx [file 12864_2024_10304_MOESM3_ESM.pdf]

Gene name	Primer name	Sequence （5‘ to 3')	Production size
TNNT1	GJ-TNNT1-F	GAAAGTGCGTATCCTGTCTGA	190
	GJ-TNNT1-R	GTGGCTGATGCGGTTGTA	
FABP3	GJ-FABP3-F	GATGAGACCACGGCAGAT	147
	GJ-FABP3-R	ATGGGTGAGTGTCAGAATGA	
TPM3	GJ-TPM3-F	CTGGCAGAGTCTAAGTGTTC	246
	GJ-TPM3-R	CTTCAGTTTCTGGGCATA	
DES	GJ-DES-F	GGAGGACCGCTTTGCTAG	284
	GJ-DES-R	TCACCGTCTTCTTGGTATGG	
RCAN1	GJ-RCAN1-F	TCAGCGAAAGTGAAACCA	218
	GJ-RCAN1-R	GTGCGAACTGCCTATGTG	
LMOD2	GJ-LMOD2-F	AACTCCTGGCTTCCCTGTC	140
	GJ-LMOD2-R	TCTGCTGAATGTCCCTGTG	
PPP1R27	GJ-PPP1R27-F	GAGTGCGTGAAGCTGCTAG	233
	GJ-PPP1R27-R	CAGTCCATCTTAGTCCCTTTG	
YWHAZ	YWHAZ-F	ACTACTATCGCTACTTGGCTGAG	87
（internal reference gene）	YWHAZ-R	CTTCTTGGTATGCTTGCTGTGA	
