# Supplementary material for: Transcriptomic and metabolomic dissection of skeletal muscle of crossbred Chongming white goats with different meat production performance
Source: BMC Genomics. 2024 May 4;25:443. doi: 10.1186/s12864-024-10304-3 (PMC11069289; doi:10.1186/s12864-024-10304-3)
Supplement: Supplementary file 4 — Additional file 4: Table S1: The primers information of RT-qPCR experiments [file 12864_2024_10304_MOESM4_ESM.pdf]

Table S1. Composition and nutrient levels of goat diets (air-dry basis) between 3-6 months

**Ingredient**	**Percentage (%)**	**Nutrient Items**	**Nutrient levels [2)]**
Corn	35.17	Metabolizable Energy /(MJ/kg)	8.42
Soybean meal	14.15	Crude Protein（%）	14.03
Corn DDGS	6.73	ether extract（%）	5. 18
Corn silage	26.03	Ash（%）	6.41
Peanut seedling	13.97	Calcium （%）	0.75
$NaHCO_3$	0.95	Phosphorus（%）	0.45
Limestone	1.27		
Ca $(HCO_3)_2$	0.31		
NaCl	0.64		
Mold remover	0.13		
Premix[1)]	0.65		
Total	100		

Table S2. Composition and nutrient levels of goat diets (air-dry basis) after 6 months

**Ingredient**	**Percentage (%)**	**Nutrient Items**	**Nutrient levels [2)]**
Corn	29.31	Metabolizable Energy /(MJ/kg)	8.04
Soybean meal	11.79	Crude Protein（%）	13.56
Corn DDGS	5.61	ether extract（%）	5. 01
Corn silage	32.54	Ash（%）	5.99
Peanut seedling	17.46	Calcium（%）	0.72
$NaHCO_3$	0.79	Phosphorus（%）	0.47
Limestone	1.06		
Ca $(HCO_3)_2$	0.26		
NaCl	0.53		
Mold remover	0. 11		
Premix [1)]	0.54		
Total	100		

Note： [1)] The premix provided the following per kg of diets： vitamin A acetate 4 640 IU , VD3 1 740 IU , DL-α-tocopheryl acetate 17. 40 mg , I 0. 41 mg , Cu 4. 64 mg , Fe 23. 20 mg , Mn 23.20 mg , Zn 29.00 mg , Se 0. 12 mg , Co 0.23 mg。 [2)] Metabolizable Energy was a calculated value, while the others were measured values.
